# Overcoming biomass recalcitrance by synergistic pretreatment of mechanical activation and metal salt for enhancing enzymatic conversion of lignocellulose

**DOI:** 10.1186/s13068-019-1354-6

**Published:** 2019-01-10

**Authors:** Yanjuan Zhang, Min Huang, Jianmei Su, Huayu Hu, Mei Yang, Zuqiang Huang, Dong Chen, Juan Wu, Zhenfei Feng

**Affiliations:** 1 School of Chemistry and Chemical Engineering, Guangxi University, Nanning, 530004 China; 2 State Key Laboratory of Non-Food Biomass and Enzyme Technology, Guangxi Academy of Sciences, Nanning, 530007 China

**Keywords:** Lignocellulose, Biomass recalcitrance, Pretreatment, Enzymatic hydrolysis, Metal salt, Mechanical activation

## Abstract

**Background:**

Due to biomass recalcitrance, including complexity of lignocellulosic matrix, crystallinity of cellulose, and inhibition of lignin, the bioconversion of lignocellulosic biomass is difficult and inefficient. The aim of this study is to investigate an effective and green pretreatment method for overcoming biomass recalcitrance of lignocellulose.

**Results:**

An effective mechanical activation (MA) + metal salt (MAMS) technology was applied to pretreat sugarcane bagasse (SCB), a typical kind of lignocellulosic biomass, in a stirring ball mill. Chlorides and nitrates of Al and Fe showed better synergistic effect with MA, especially AlCl_3_, ascribing to the interaction between metal salt and oxygen-containing groups induced by MA. Comparative studies showed that MAMS pretreatment effectively changed the recalcitrant structural characteristics of lignocellulosic matrix and reduced the inhibitory action of lignin on enzymatic conversion of SCB. The increase in hydroxyl and carboxyl groups of lignin induced by MAMS pretreatment led to the increase of its hydrophilicity, which could weaken the binding force between cellulase and lignin and reduce the nonproductive binding of cellulase enzymes to lignin.

**Conclusions:**

MAMS pretreatment significantly enhanced the enzymatic digestibility of polysaccharides substrate by overcoming biomass recalcitrance without the removal of lignin from enzymatic hydrolysis system.

**Electronic supplementary material:**

The online version of this article (10.1186/s13068-019-1354-6) contains supplementary material, which is available to authorized users.

## Background

Recently, the conversion of lignocellulosic biomass into biofuels and chemicals has garnered extensive interest and is considered as a viable technical scheme, and biomass-derived sugars have been proposed as intermediates for the production of biofuels or chemicals [1]. Lignocellulosic biomass is the most abundant and renewable source on earth for producing second-generation bioethanol and other value-add chemicals through the sugar platform [2], and the typical process for deconstructing lignocellulose to sugars is enzymatic hydrolysis [3, 4]. Lignocellulosic biomass is mainly composed of cellulose, hemicellulose, and lignin. Cellulose and hemicellulose are chain polysaccharides, while lignin is a heterogeneous and cross-linked three-dimensional phenyl-propane polymer. These components are strongly intermeshed and bonded through covalent or non-covalent bonds, forming the complex and recalcitrant lignocellulosic matrix [5].

The molecular chains of cellulose have a tendency to form intra- and intermolecular hydrogen bonds through hydroxyl groups, which promote cellulose aggregations and lead to a supramolecular structure with high degree of polymerization (DP) and crystallinity [6]. The strong interchain hydrogen bonding networks make crystalline cellulose resistant to enzymatic digestibility, whereas hemicellulose and amorphous cellulose are readily digestible [7]. The structural characteristics of lignocellulosic biomass form strong native recalcitrance to enzymes and result in relatively low saccharification efficiency [3, 8]. Therefore, various pretreatment methods have been widely studied and developed to break the recalcitrant native structure of lignocellulose and the stable crystal structure of cellulose [9, 10].

Lignin can inhibit the enzymatic hydrolysis of cellulose through physical blockages, such as hydrophobicity, surface charges, and hydrogen bonding interactions, which limit the accessibility of polysaccharides substrate and nonproductive binding of cellulase enzymes to lignin [11, 12]. To improve the activity of enzymes and achieve high saccharification yield from lignocellulosic biomass, it is critical to reduce the nonproductive adsorption of enzymes [13]. Complete removal of lignin by further delignification is not only expensive but also unnecessary to enhance the saccharification of polysaccharides substrate. For common pretreatments of lignocellulosic biomass without effective delignification, nonproductive binding of enzymes is unavoidable in the enzymatic saccharification of lignocellulose, and higher enzymes loading is required to achieve desired saccharification efficiency [14]. The addition of surfactant and protein has been reported for reducing the nonproductive binding of enzymes on lignin by changing the structure of substrate [14, 15]. For this purpose, it is significant to develop an appropriate pretreatment for changing the structure of lignin to reduce its inhibitory action and simultaneously destroy the recalcitrant structure of lignocellulose and highly ordered crystalline structure of cellulose, which can efficiently overcome biomass recalcitrance.

Mechanical activation (MA), the use of intense milling to change the structures and physicochemical properties of solid materials without the use of solvents, intermediate fusion, etc., can lead to size reduction and structural disorder of the solids accompanied by distorting and rupture of chemical bonds [16]. MA has been used as a simple, effective, and environmentally friendly physical pretreatment to destroy the recalcitrant structure of lignocellulose and stable crystal structure of cellulose, and change the chemical structures of lignin [17–20]. Metal salts can be used for the pretreatment of cellulosic materials by disrupting the hydrogen bonds, reducing the crystallinity of cellulose, and aiding in the cleavage of glycosidic linkages, which result in the enhancement of enzymatic digestibility [21]. Metals salts are especially fascinating as pretreatment agents because of their lower corrosivity than inorganic acids [22]. Typical and common metal salts, such as chlorides, nitrates, and sulfates of Al, Fe, Mg, and K, are usually chosen for the pretreatment of lignocellulosic biomass [23, 24]. Because of the poor contact between metal salts and cellulosic materials, metal salt pretreatment cannot be performed in solid-phase condition but in molten state or aqueous solution [25]. As the assistance of MA, intense milling can make metal salt easily move and contact with cellulosic materials in solid phase. Therefore, a combined technology of synergistic interaction of MA + metal salt (MAMS) in solid-phase condition has been developed for the pretreatment of cellulose, which could induce more effective changes in structural characteristics of cellulose and significantly enhance the enzymatic saccharification of cellulose [26]. It is significant to investigate how MAMS pretreatment disrupts the recalcitrant structure of lignocellulosic biomass and changes the structure of lignin, which may affect the enzymatic conversion of lignocellulose.

Sugarcane bagasse (SCB), a major agro-industrial residue, is a typical kind of lignocellulosic biomass. This material could be effectively utilized for producing bioethanol or other chemicals by biorefinery [27, 28]. The enzymatic conversion of lignocellulosic biomass is a complicated process and the main influencing factors have been found to be structural features of lignocellulose, including chemical compositions and physical structure. In the present study, effective metal salts were first chosen by comparing the saccharification yield of different metal salt + MA-pretreated SCB samples, and then the structural characteristics of the SCB samples pretreated by MA and MAMS were measured to comparatively investigate the effects of structure changes on enzymatic saccharification of SCB. Moreover, holocellulose (cellulose + hemicellulose) and lignin were isolated from SCB to comparatively investigate the effect of MAMS pretreatment on the reactivity and accessibility of polysaccharides substrate without the blockage of lignin and evaluate the nonproductive binding of enzymes on lignin, respectively.

## Results and discussion

### Effect of different metal salts on the enzymatic hydrolysis of SCB

It has been reported that only MA pretreatment could not effectively enhance the enzymatic saccharification of cellulose, and a synergistic interaction of MA and metal salt treatment induced more effective changes in the structural characteristics of cellulose and thus led to significant increase in the enzymatic hydrolysis efficiency of cellulose [26]. Different from cellulose, lignocellulose is composed of compact cellulose–hemicellulose–lignin complex, so the pretreatment that could effectively improve the enzymatic digestibility of lignocellulose should destroy its recalcitrant structure and reduce the crystallinity of cellulose. In addition, for the pretreatment without delignification, it is important to overcome the blockage of lignin and reduce nonproductive binding of enzymes to lignin, which contribute to the increase in the accessibility of cellulose and the activity of enzyme.

**Fig. 1:**
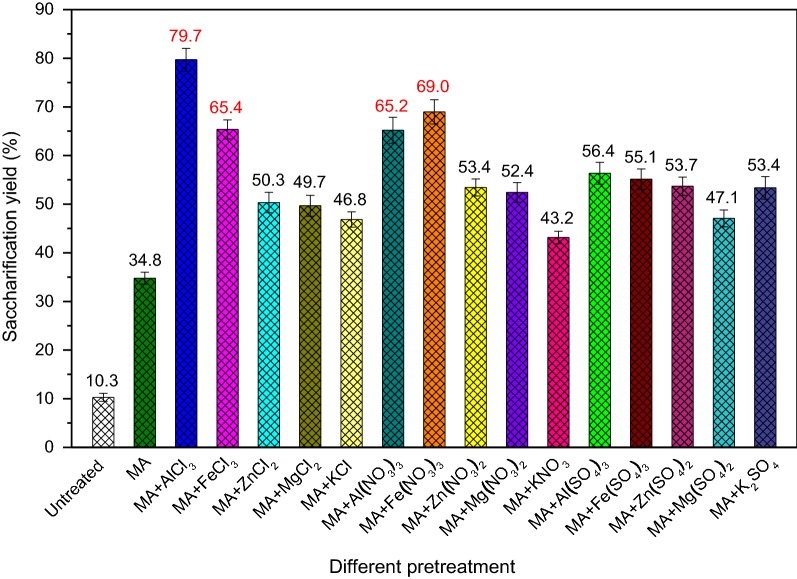
Saccharification yields of untreated and different pretreated SCB samples

To investigate the synergistic deconstructive cooperation between MA and metal salt, a range of typical and common metal salts, including different metal chlorides, nitrates, and sulfates, were applied as additives for the pretreatment of lignocellulose. The different metal salt + MA-pretreated SCB was hydrolyzed by cellulase enzymes, and the results are presented in Fig. 1. The saccharification efficiencies of untreated and MA-pretreated SCB samples were 10.3% and 34.8%, respectively. By contrast, the presence of metal salt had positive effect on improving the saccharification efficiency of lignocellulose, especially the chlorides and nitrates of trivalent metal ions, i.e., AlCl_3_, FeCl_3_, Al(NO_3_)_3_, and Fe(NO_3_)_3_, with the saccharification yield of 79.7%, 65.4%, 65.2%, and 69.0%, respectively. It is of significance to explore the mechanism that how these four effective metal salts combined with MA affect the enzymatic hydrolysis of lignocellulose.

During the process of MAMS, continuous impacts and collisions induced by milling balls could impart a significant amount of mechanical energy to the system, which could cause the homolytic bond cleavage of AlCl_3_ and FeCl_3_ (covalent compounds) to form free radicals [29, 30], i.e., Al·, Fe·, and Cl·. Al· and Fe· have great tendency to compete the covalent electron of the oxygen of hydroxyl groups, while Cl· has great tendency to compete the covalent electron of the hydrogen of hydroxyl groups. For Al(NO_3_)_3_ and Fe(NO_3_)_3_ (ionic compounds), Al^3+^ and Fe^3+^ are the highly oxophilic cations, which could easily form a coordination bond with the oxygen of hydroxyl groups. In addition, NO_3_^−^ and Cl^−^ anions had great tendency to combine with the hydrogen of hydroxyl groups, so these anions provided a stoichiometric amount of hydrogen bonding to the hydroxyl groups of lignocellulose [31]. Effective destruction of the recalcitrant structure of lignocellulose and the reduction of high crystallinity of cellulose mainly depend on the direct interaction of their hydroxyl groups with free radicals or ions of metal salts, leading to the destruction of inter- and intramolecular hydrogen bonds in lignocellulose. Because of the better oxophilicity of Al· and better electronic capture capability of Cl·, AlCl_3_ exhibited the best effect among these four metal salts. Furthermore, instantaneous high temperature induced by intense milling could lead to the melting of metal salt [32], which increased the fluidity and permeability of metal salt in lignocellulose. As a result, the metal salt with lower melting point temperature exhibited better synergistic interaction with MA. The melting point of AlCl_3_, FeCl_3_, Al(NO_3_)_3_, and Fe(NO_3_)_3_ was 190, 306, 74, and 47 °C, respectively. Although FeCl_3_ could form highly active Fe· and Cl·, its high melting point led to relatively poor effect on enzymatic hydrolysis of SCB compared with that of AlCl_3_. By contrast, Fe(NO_3_)_3_ with a low melting point showed better effect than that of Al(NO_3_)_3_ and FeCl_3_. To further confirm the mechanism of MAMS pretreatment for enhancing the enzymatic conversion of lignocellulosic biomass, the structural characteristics of the SCB samples pretreated by MA + effective metal salts (AlCl_3_, FeCl_3_, Al(NO_3_)_3_, and Fe(NO_3_)_3_) were systematically studied.

### Changes in the structural characteristics of SCB with different pretreatments

**Fig. 2:**
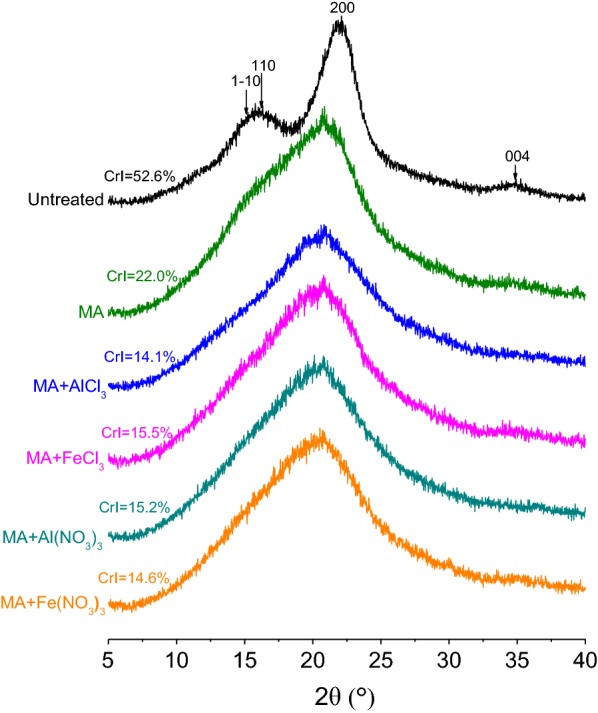
XRD patterns of untreated and different pretreated SCB samples

The crystallinity of cellulose is believed to result in the poor accessibility and low enzymatic digestibility. As cellulose is the most critical component in lignocellulose, the crystallinity is widely measured and related to the enzymatic conversion of lignocellulose [33]. Crystallinity index (CrI) is a parameter used to describe a qualitative or semi-quantitative evaluation of the amounts of amorphous and crystalline cellulosic components in a sample [34]. X-ray diffraction (XRD) analysis provides direct information relating to the crystal and amorphous parts of cellulose, and CrI of cellulosic materials can be evaluated by XRD analysis [35]. XRD patterns of native and different pretreated SCB samples are illustrated in Fig. 2.

Untreated SCB shows remarkable crystal structure as the strong peaks at 2*θ* about 14°–17°, 21°–22°, and 34.8°, corresponding to 1–10, 110, and 200 planes of crystalline cellulose I [36]. SCB contains a certain amount of amorphous lignin and hemicellulose, which contribute to the relatively weaker diffraction of 1–10, 110, and 004 planes and the overlap of 1–10 and 110 planes compared with pure cellulose [18, 37]. Clearly, intense milling caused the destruction of ordered crystal structure and macromolecular chains of cellulose, leading to a significant decrease in the intensity of the characteristic peaks of crystalline cellulose. With the presence of metal salt in ball milling, the crystal structure of cellulose was damaged more seriously. The CrI of untreated SCB was 52.6%, but decreased to 22.0%, 14.1%, 15.5%, 15.2%, and 14.6% after MA, MA + AlCl_3_, MA + FeCl_3_, MA + Al(NO_3_)_3_, and MA + Fe(NO_3_)_3_ pretreatment, respectively. MAMS pretreatment exhibited outstanding effect on breaking the crystal structure of cellulose in SCB, demonstrating the synergistic interaction of MA and metal salt. The outer layer of lignocellulose was first disrupted by MA to provide easy access for metal salt to the interior for further action of MA and metal salt on lignocellulose, attributing to a remarkable reduction in the crystallinity of cellulose. The crystallinity of lignocellulosic biomass has been regarded as a major factor that influences enzymatic hydrolysis [38], so the pretreated SCB with lower CrI exhibited higher saccharification yield. Moreover, it can be noticed that the saccharification yield of pretreated SCB was not linearly proportional to the CrI, especially the MAMS-pretreated SCB with similar CrI had different saccharification yields, implying that the destruction of crystal structure of cellulose is not the only major influencing factor. Therefore, further investigations should be performed to analyze other factors that affect the saccharification efficiency of SCB.

**Fig. 3:**
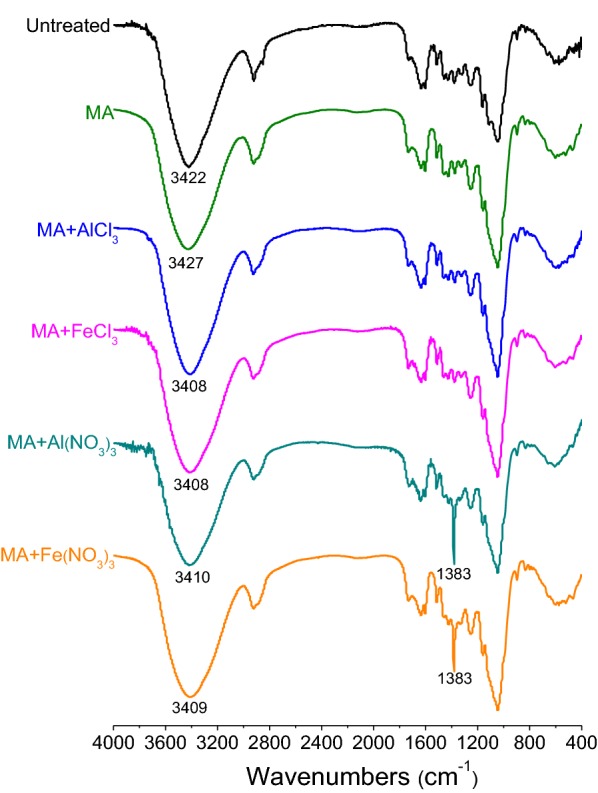
FTIR spectra of untreated and different pretreated SCB samples

Fourier transform infrared spectroscopy (FTIR) analysis can reveal the functional groups and chemical structure of the SCB samples before and after pretreatment. FTIR spectra of different samples are presented in Fig. 3. All the spectra show characteristic absorption peaks of lignocellulose. After MA + Al(NO_3_)_3_ or MA + Fe(NO_3_)_3_ pretreatment, a new sharp adsorption band at 1383 cm^−1^ can be observed, which is assigned to the characteristic absorption peak of nitrate (NO^3−^) anion [39]. No other new absorption peaks appear in the spectra of pretreated SCB samples. A broad absorption peak at 3422 cm^‒1^ in the spectrum of untreated SCB is attributed to the stretching vibration of –OH groups [40]. After MA pretreatment, this absorption peak shifted to a higher wavenumber of 3427 cm^−1^, indicating that MA enhanced internal energy and reactivity of SCB by the destruction of inter- and intramolecular hydrogen bonds and the increase of free hydroxyl groups [17]. However, in the spectra of MAMS-pretreated SCB, the absorption peak of –OH groups shifted to a lower wavenumber compared with that of untreated and MA-pretreated SCB, implying a strong evidence for the interaction between the hydroxyl groups and metal salts induced by intense milling [41]. As a result, the hydrogen bonds were further deconstructed by MAMS, leading to the reduction in crystallinity and the improvement in the enzymatic hydrolysis of SCB, which accords with the results of enzymatic saccharification and XRD analysis of different pretreated SCB samples.

X-ray photoelectron spectroscopy (XPS) analysis was performed to investigate the element composition and the surface chemical bonding of the samples (Additional file 1: Fig. S1). The survey spectra (Additional file 1: Fig. S1a) show that the main elements of all the samples were C and O, and the elements of the metal salts used for pretreatment were also determined. The elemental compositions in atomic percentage derived from XPS survey spectra are presented in Table 1, which shows that the atomic ratio of O/C in untreated SCB was 0.276, but increased to 0.414, 0.598, 0.591, 0.606, and 0.598 after pretreated by MA, MA + AlCl_3_, MA + FeCl_3_, MA + Al(NO_3_)_3_, and MA + Fe(NO_3_)_3_, respectively. This indicates that pretreatment could effectively destroy the hydrogen bonds and active oxygen-containing groups exposed on the surface of SCB, especially by MAMS pretreatment, showing the synergistic interaction between MA and metal salts. The high-resolution C 1 s and O 1 s XPS spectra provided detailed information regarding the surface chemistry (Additional file 1: Fig. S1b and c), and the deconvolution results of C 1 s and O 1 s regions are shown in Table 2. The C 1 s spectra were deconvoluted into three peaks, corresponding to C1 (C–H, C–C), C2 (C–O, C–O–C), and C3 (C=O, O=C–O) [41], which show that the fraction of C1 decreased, while that of C2 and C3 increased after pretreatment. The increase of C3 may be ascribed to the oxidation of active oxygen-containing groups induced by pretreatment, especially MAMS pretreatment. The O 1 s spectra were curve fitted into O1 (C=O) and O2 (C–OH, C–O–C) [42], showing that the fraction of O1 increased with that of C3, and the fraction of O2 which further confirmed the generation of aldehyde or carboxyl groups (C=O/O=C–O) induced by the oxidation of active oxygen-containing groups during the process of pretreatment. Therefore, MA and MAMS pretreatments evidently affected the surface chemical bonding, contributing to the changes in the structural characteristics of SCB, which could help to promote its enzymatic conversion.

**Table 1:** Elemental compositions in atomic percentage derived from XPS survey spectra

Sample	Atomic percentage (%)						O/C
	O 1 s	C 1 s	Al 2p	Fe 2p	Cl 2p	N 1 s	
Untreated SCB	21.61	78.39	–	‒	‒	‒	0.276
MA-pretreated SCB	29.30	70.70	‒	‒	‒	‒	0.414
MA + AlCl_3_ pretreated SCB	36.43	60.93	1.94	‒	0.69	‒	0.598
MA + FeCl_3_ pretreated SCB	36.49	61.71	‒	0.96	0.84	‒	0.591
MA + Al(NO_3_)_3_ pretreated SCB	36.24	59.82	1.69	‒	‒	2.25	0.606
MA + Fe(NO_3_)_3_ pretreated SCB	36.08	60.36	‒	0.64	‒	2.92	0.598

**Table 2:** Relative fractions of the C and O contributions derived from peak fitting of high-resolution C 1 s and O 1 s XPS spectra

Sample	Relative fraction of C (%)			Relative fraction of O (%)	
	C1	C2	C3	O1	O2
Untreated SCB	69.04	25.02	5.94	19.19	81.81
MA-pretreated SCB	56.65	33.20	10.15	20.95	79.05
MA + AlCl_3_ pretreated SCB	36.70	43.70	16.00	25.37	74.63
MA + FeCl_3_ pretreated SCB	37.08	48.21	14.71	23.37	76.63
MA + Al(NO_3_)_3_ pretreated SCB	36.42	48.14	15.43	23.95	76.05
MA + Fe(NO_3_)_3_ pretreated SCB	36.63	47.82	15.55	24.64	75.36

**Fig. 4:**
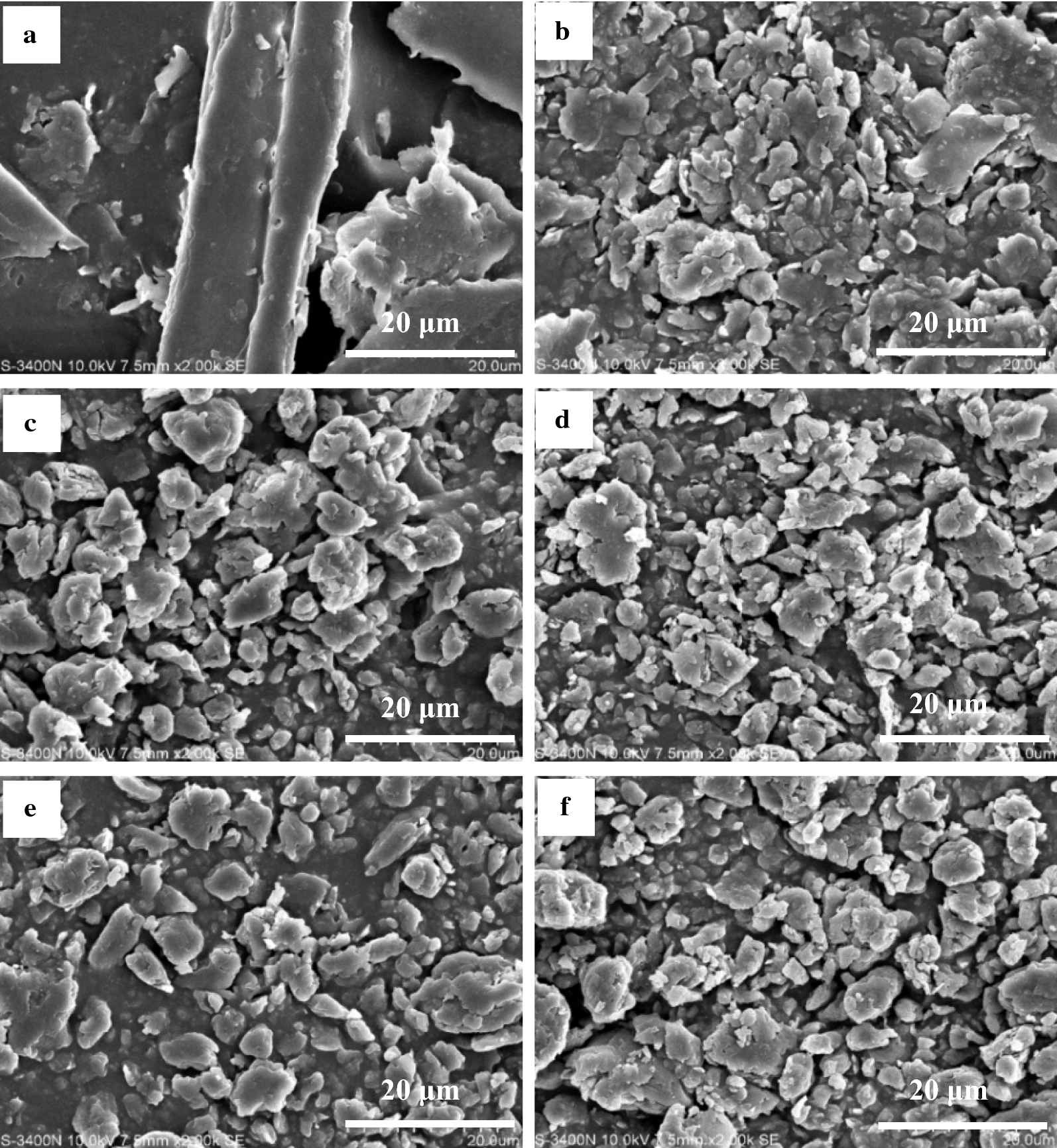
SEM micrographs of **a** untreated SCB and the SCB samples pretreated by different methods: **b** MA, **c** MA + AlCl_3_, **d** MA + FeCl_3_, **e** MA + Al(NO_3_)_3_, and **f** MA + Fe(NO_3_)_3_

Scanning electron microscopy (SEM) is a powerful tool that is widely used to investigate the surface of lignocellulose, including surface characterization, morphology, and analysis of microstructure. Although SEM analysis is mainly qualitative, it can provide us with an insight into the microstructure for directly observing the damage to surface morphology of lignocellulose by different pretreatments. The morphological changes were explored by comparing SEM images of untreated and different pretreated SCB samples (Fig. 4). The changes in morphology of the MA and MAMS-pretreated SCB were similar, indicating that the destruction of morphological structure of SCB was mainly attributed to ball milling. Clearly, the pretreatment resulted in the materials with smaller particles, rougher surface, more folds and cracks, and increased surface area. The damage of the physical structure during pretreatment has an important influence on reducing biomass recalcitrance. It is generally accepted that the presence of lignin in plant cell walls physically impedes the accessibility of enzymes to polysaccharides substrate [43]. During the process of MA or MAMS pretreatment, intense milling could lead to the isolation, migration, and redistribution of lignin, which was one of the responsible events for opening up the compact packed matrix. Compared with untreated SCB, the pretreated samples could be more accessible for hydrolytic enzymes to the interior of polysaccharides substrate, resulting in higher saccharification yields. Therefore, both MA and MAMS pretreatments significantly altered the compact structure of SCB and thus decreased the recalcitrance from its physical structure.

### Comparison of the enzymatic hydrolysis of SCB and holocellulose by different pretreatments

**Fig. 5:**
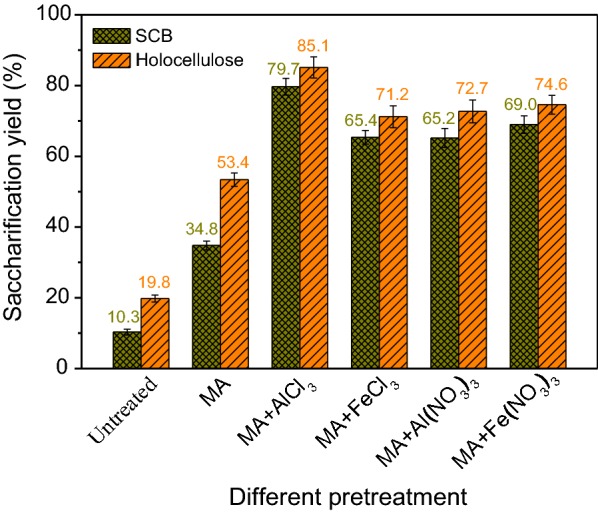
Saccharification yields of untreated and different pretreated holocellulose and SCB samples

Lignin is considered as the most recalcitrant component in biopolymers for physically impeding the accessibility of enzymes to polysaccharides substrate, and the effect of lignin on the enzymatic digestibility of lignocellulosic biomass has received extensive attention [11, 44]. In general, lignin content is negatively correlated with the bioavailability of polysaccharides for enzymatic saccharification and further fermentation to produce bioethanol, which mainly from two aspects. One is that lignin acts as a physical barrier to restrict the access of enzymes to polysaccharides substrate, and the other is that lignin can irreversibly adsorb enzymes to prevent their actions [43]. To evaluate the effect of lignin on inhibiting the enzymatic digestibility of lignocellulosic biomass, holocellulose was isolated from SCB by delignification, and the enzymatic hydrolysis of the different pretreated SCB and holocellulose samples was comparatively investigated. As shown in Fig. 5, saccharification yields of all the holocellulose samples were higher than those of the corresponding SCB samples, showing that delignification effectively increased the enzymatic digestibility of polysaccharides. As isolating lignin from SCB, the physical barrier of lignin and the nonproductive adsorption of enzymes on lignin were virtually eliminated, implying a direct relationship between the presence of lignin and saccharification efficiency of lignocellulose. In addition, the influences of different pretreatments on the enzymatic digestibility of SCB and holocellulose were mainly similar. The DP and CrI of cellulose were remarkably decreased by MAMS pretreatment and thus efficiently enhanced the enzymatic hydrolysis of holocellulose [26].

However, MA pretreatment was more effective for enhancing the enzymatic hydrolysis of holocellulose than that of SCB, and the increment in saccharification yields of MAMS-pretreated SCB samples was more than that of MAMS-pretreated holocellulose samples compared with the corresponding untreated and MA-pretreated ones. This indicates that the interaction of MA and metal salt had special effect on the cross-linkage structure of lignin-polysaccharides and the structure changes of lignin, and delignification greatly benefitted to improve the enzymatic hydrolysis of lignocellulose but not the only prerequisite for enhancing the accessibility of polysaccharides substrate. In most case, it is actually too complicated and expensive to be feasible for delignification of lignocellulosic biomass [11]. Applying a suitable pretreatment to dramatically reduce the inhibitory action of lignin to enzymatic digestibility of polysaccharides without the removal of lignin from lignocellulose is economically feasible and relatively easier to operate. The results in Fig. 5 show that the saccharification yield of MA + AlCl_3_ pretreated SCB reached a high value and approached to that of MA + AlCl_3_ pretreated holocellulose, demonstrating that the effective MAMS pretreatment could avoid delignification of lignocellulose and obtain high saccharification yields. To understand how MAMS pretreatment reduced the influence of lignin on enzymatic digestibility of polysaccharides substrate, the isolated lignin was pretreated by different methods and then was added to the enzymatic hydrolysis system of holocellulose to investigate the changes in saccharification yield, and the structure changes of corresponding pretreated lignin were also studied.

### Effect of different pretreated lignin on the enzymatic hydrolysis of holocellulose

**Fig. 6:**
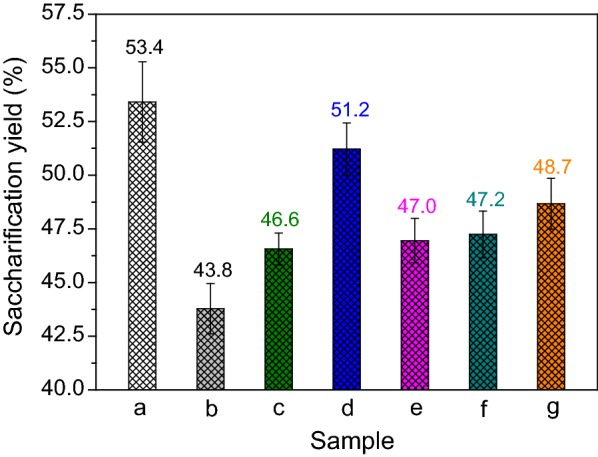
Saccharification yields of **a** MA-pretreated holocellulose and **a** + different pretreated lignin: **b** untreated, **c** MA, **d** MA + AlCl_3_, **e** MA + FeCl_3_, **f** MA + Al(NO_3_)_3_, and **g** MA + Fe(NO_3_)_3_

The isolation of lignin from lignocellulose has been proved to efficiently enhance the enzymatic hydrolysis of polysaccharides substrate, and other lignin-related prominent factors impacting the biomass digestibility may include the chemical structures of lignin [6]. For this reason, the lignin isolated from SCB was used as untreated lignin and then was pretreated by MA and MAMS, and then the saccharification yields of MA-pretreated holocellulose with the addition of untreated and different pretreated lignin samples (the adding amount of lignin was in accordance with its abundance ratio in SCB). As presented in Fig. 6, the saccharification yield of the MA-pretreated holocellulose without lignin was the highest, and the addition of any kind of lignin samples reduced the saccharification yield of holocellulose, especially the untreated lignin. This result strongly confirms that the presence of lignin impeded full action of enzymes on polysaccharides substrate.

By comparatively analyzing the data in Figs. 5 and 6, the effects of the nonproductive adsorption of cellulase on lignin and the hindrance of compact lignocellulosic matrix on enzymatic hydrolysis efficiency of biomass also could be approximately determined. The saccharification yields of MA-pretreated holocellulose, MA-pretreated holocellulose + untreated lignin, and MA-pretreated SCB were 53.4%, 43.8%, and 34.8%, respectively, suggesting that both nonproductive adsorption of enzymes and complex cross-linkage structure of lignin-polysaccharides affected the enzymatic hydrolysis of polysaccharides substrate, and the influence of the latter was greater than that of the former. As lignin had been isolated from SCB, it could accurately investigate the effects of different pretreatments of lignin on reducing the nonproductive binding of enzymes to lignin from Fig. 6 without considering the hindrance of the compact cellulose-hemicellulose-lignin matrix. With the addition of untreated lignin to the enzymatic hydrolysis system of holocellulose, the inhibitory action of lignin led to the decrease of saccharification yield of holocellulose from 53.4 to 43.8%. After pretreated by MA or MAMS, the inhibitory action of lignin evidently decreased. Especially for adding the MA + AlCl_3_ pretreated lignin, the saccharification yield of holocellulose could reach 51.2%, which was close to that of pure holocellulose, implying that MAMS pretreatment of lignin could effectively reduce its inhibition to enzymatic catalysis. Therefore, MAMS pretreatment could be considered as an effective and simple method to destroy the recalcitrant structure of lignocellulosic matrix and crystal structure of cellulose and reduce the inhibitory action of lignin in biomass digestibility without the removal of lignin from enzymatic hydrolysis system.

### Changes in the chemical structures of lignin with different pretreatments

**Fig. 7:**
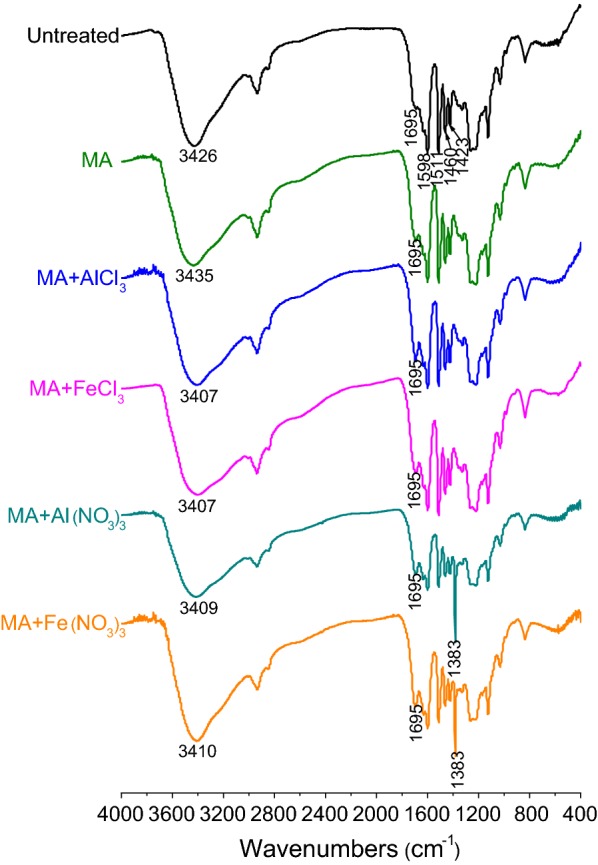
FTIR spectra of untreated and different pretreated lignin samples

It has been indicated that the structure of lignin plays a significant role in the inhibition of cellulose hydrolysis [45]; therefore, the investigation of the structure changes of the lignin after different pretreatments can help to expound that the inhibition of pretreated lignin significantly lower than that of untreated one. Lignin contains a large number of functional groups, including aliphatic and phenolic hydroxyls, carboxyl, carbonyl and methoxyl groups, conjugated double bonds, etc., which have direct effect on its special properties [46]. FTIR analysis can indicate the presence of functional groups in different lignin samples and confirm the interaction between lignin and metal salts promoted by ball milling. FTIR spectra of different lignin samples are presented in Fig. 7. All the spectra show the characteristic peaks at 1598, 1511, 1460, and 1423 cm^‒1^, corresponding to aromatic skeleton vibrations [47], which confirmed that this isolated component was lignin. After MA + Al(NO_3_)_3_ or MA + Fe(NO_3_)_3_ pretreatment, the characteristic absorption peak of nitrate (NO^3−^) anion appeared at 1383 cm^−1^. The stretching vibration of –OH groups in the spectrum of untreated lignin presented at 3426 cm^‒1^, but shifted to a higher wavenumber of 3435 cm^−1^ after MA pretreatment, indicating that MA enhanced internal energy and reactivity of lignin. However, the absorption peak of –OH groups in the spectra of MAMS-pretreated lignins shifted to a lower wavenumber compared with that of untreated and MA-pretreated lignins, implying the interaction between the hydroxyl groups and metal salts induced by intense milling. In addition, a peak at 1695 cm^‒1^ corresponding to carbonyl groups became stronger after pretreatment, especially MAMS pretreatment, which may be due to the oxidation of oxygen-containing groups and the generation of aldehyde, ketone, or carboxyl groups [17], implying that the combination of MA and metal salt significantly enhanced the reactivity of lignin. The change in the functional groups of lignin could weaken the adverse effect of lignin on the enzymatic digestibility of polysaccharides.

XPS analysis was also performed to investigate the surface chemical bonding of lignins (Additional file 1: Fig. S2). The survey XPS spectra (Additional file 1: Fig. S2a) show that the main elements of all the samples were C and O, and the elements of the metal salts used for pretreatment were also determined. The elemental compositions in atomic percentage derived from XPS survey spectra are presented in Table 3, which shows that the atomic ratio of O/C in MA and MAMS-pretreated lignins increased by a certain amount compared with that of untreated lignin, but the increment was less than that of the pretreated SCB samples. This may be due to that strong hydrogen bonds of crystalline cellulose in SCB were effectively destroyed by pretreatment and generated free hydroxyl groups on the surface of SCB, but lignin is an amorphous polymer without strong hydrogen bonds, so the change in atomic ratio of O/C before and after pretreatment was not so significant. The deconvolution results of C 1 s and O 1 s regions are shown in Additional file 1: Fig. S2b and c and Table 4. The C 1 s spectra were deconvoluted into three peaks [41], and the fraction of C1 (C–H, C–C) decreased, while that of C2 (C–O, C–O–C) and C3 (C=O, O=C–O) increased after pretreatment. The increase of C3 could be ascribed to the oxidation of oxygen-containing groups induced by pretreatment, especially MAMS pretreatment, which was in good agreement with FTIR analysis. The O 1 s spectra were deconvoluted into two peaks [42], and the fraction of O1 (C=O) increased with that of C3. The fraction of O2 (C–OH, C–O–C) decreased, which further confirmed the oxidation of O2 to O1 during pretreatment, especially MA + AlCl_3_ pretreatment. As a result, the surface chemical bonding of lignin changed by pretreatment could change the effect of lignin on the enzymatic digestibility of lignocellulosic biomass.

**Table 3:** Elemental compositions in atomic percentage derived from XPS survey spectra

Sample	Atomic percentage (%)						O/C
	O 1 s	C 1 s	Al 2p	Fe 2p	Cl 2p	N 1 s	
Untreated lignin	22.67	77.33	‒	‒	‒	‒	0.293
MA-pretreated lignin	25.94	74.06	‒	‒	‒	‒	0.350
MA + AlCl_3_ pretreated lignin	25.04	73.46	1.14	‒	0.36	‒	0.341
MA + FeCl_3_ pretreated lignin	24.48	72.02	‒	0.98	0.52	‒	0.331
MA + Al(NO_3_)_3_ pretreated lignin	24.83	71.51	1.36	‒	‒	2.30	0.347
MA + Fe(NO_3_)_3_ pretreated lignin	26.28	69.65	‒	0.76	‒	3.39	0.377

**Table 4:** Relative fractions of the C and O contributions derived from peak fitting of high-resolution C 1 s and O 1 s XPS spectra

Sample	Relative fraction of C (%)			Relative fraction of O (%)	
	C1	C2	C3	O1	O2
Untreated lignin	66.99	31.12	1.89	5.66	93.34
MA-pretreated lignin	60.55	36.33	3.12	8.09	91.91
MA + AlCl_3_ pretreated lignin	60.61	33.94	5.45	14.16	85.84
MA + FeCl_3_ pretreated lignin	60.94	34.98	4.08	10.15	89.85
MA + Al(NO_3_)_3_ pretreated lignin	60.45	35.31	4.24	10.87	89.13
MA + Fe(NO_3_)_3_ pretreated lignin	59.98	35.12	4.90	12.28	87.72

Ultraviolet/visible (UV/vis) spectroscopy is a convenient and useful tool for qualitative and quantitative analyses of lignin in solution attributing to that the characteristic aromatic rings of lignin result in a strong UV absorption at approximately 300–380 nm, from which the free phenolic hydroxyls can be quantified [48]. Herein, neutral spectrum was used to analyze the type of lignin, and ionization difference spectrum was used for estimating the content of free phenolic hydroxyl groups (Additional file 1: Fig. S3). As shown in Additional file 1: Fig. S3a, the maximum absorption peaks in the neutral spectra of lignin did not shift after pretreatment, indicating that MA or MAMS did not alter the type of lignin. The ionization difference spectra of untreated and pretreated lignins are shown in Additional file 1: Fig. S3b, and the main absorption peaks in all the spectra are at the same frequency, proving the same type of phenolic hydroxyl in these lignin samples. As the absorbance obeys the Beer–Lambert law, the content of free phenolic hydroxyl groups could be determined from the difference spectra using the following equation [49]:

1 $$q = \frac{{\Delta \alpha_{\text{max} } }}{\Delta \varepsilon } \times 100\%$$

where Δ*α*_max_ (l g^−1^ cm^−1^) is the difference in absorptivity between ionized and non-ionized compounds, and Δ*ε* (l mol^−1^ cm^−1^) is the difference of molar absorptivity.

As presented in Additional file 1: Fig. S3b, the difference spectra show their Δ*α*_max_ at approximately 300 and 370 nm. According to Lin [48], these wavelengths correspond to type I and type IV phenolic structures, respectively, which can be calculated using Eq. (1). Δ*ε* is the same for all the same type of lignin, *q* ∝ Δ*α*_max_. So Δ*α*_max_ can be directly used to estimate the content of phenol hydroxyl groups. The Δ*α*_max_ at 300 and 370 nm of untreated and different pretreated lignins were measured from the UV/vis spectra and the values are presented in Table 5, indicating that the content of phenolic hydroxyl groups significantly increased after pretreatment, and the interaction of MA and AlCl_3_ exhibited greater effect.

**Table 5:** Δ*α*_max_ at 300 and 370 nm of different lignin samples from UV/vis spectra

Sample	Δ*α*_max_ at 300 nm	Δ*α*_max_ at 370 nm
Untreated lignin	0.01	1.24
MA-pretreated lignin	4.57	4.15
MA + AlCl_3_ pretreated lignin	13.46	10.28
MA + FeCl_3_ pretreated lignin	4.91	4.87
MA + Al(NO_3_)_3_ pretreated lignin	6.07	5.49
MA + Fe(NO_3_)_3_ pretreated lignin	9.85	8.26

^1^H NMR was also used for investigating the detailed chemical structure of lignin (Additional file 1: Fig. S4). Trioxane was used as internal standards in order to study the change in the content of functional groups. The peaks at 1.1–1.4, 2.8–3.5, 3.5–4.3, 6.2–8.0, 10.0–10.2, and 12.1–12.4 ppm correspond to alkyl, aliphatic hydroxyl (AlOH), methoxyl (–OCH_3_), aromatic H (ArH), phenolic hydroxyl (ArOH), and carboxyl (COOH), respectively. The peaks of AlOH and –OCH_3_ overlapped to form a broad peak, which could be separated by deconvolution curve fitting. The integrated area of trioxane (4.9–5.4 ppm) was chosen as reference, the integrated area of AlOH, –OCH_3_, ArOH, and COOH were calculated, and the results are presented in Table 6.

**Table 6:** Integration of the functional groups of lignin samples from ^1^H NMR spectra

Sample	AlOH	–OCH_3_	ArOH	COOH	CH_2_ of trioxane
Untreated lignin	2.16	1.87	0.05	0	1.00
MA-pretreated lignin	2.55	1.59	0.08	0.08	1.00
MA + AlCl_3_ pretreated lignin	2.94	1.17	0.15	0.12	1.00
MA + FeCl_3_ pretreated lignin	2.70	1.42	0.11	0.09	1.00
MA + Al(NO_3_)_3_ pretreated lignin	2.83	1.31	0.12	0.09	1.00
MA + Fe(NO_3_)_3_ pretreated lignin	2.87	1.25	0.13	0.11	1.00

Comprehensive analyses of UV/vis and ^1^H NMR show that the contents of aliphatic and phenolic hydroxyl groups increased after pretreatment, particularly MA + AlCl_3_ pretreatment, but the content of methoxyl groups decreased. In the process of ball milling, the instantaneous high temperature may cause a series of free radical reaction, and the demethylation and depolymerization/repolymerization were took place simultaneously, attributing to the formation of aliphatic and phenolic hydroxyl groups and the consumption of methoxyl groups. Moreover, oxygen might cause partial oxidation of aldehyde to carboxyl groups as lignin directly exposed to air, leading to a slight increase in the content of carboxyl groups after MA [19]. With the addition of metal salt, the promoting effect on these reactions was more significant.

Enzymes bind to lignin through hydrophobic, electrostatic, and hydrogen bonding interactions. The lignin backbone is hydrophobic, and hydrophobic interaction is considered to be the primary driving force for protein adsorption [11, 14, 50]. After MA or MAMS pretreatment, the surface of lignin became more hydrophilic because of the increased amount of hydroxyl and carboxyl groups, which have less affinity to enzymes. The increase in hydrophilicity of lignin weakened the binding force between enzymes and lignin. In addition, metal ions could combine with lignin to form lignin-metal complexes, which helped to decrease the binding sites of lignin to enzymes by electrostatic or hydrogen bonding interaction, showing that the presence of metal salt could promote the enzymatic hydrolysis of lignocellulose [51, 52]. As a result, the concentration of free enzymes in the suspension increased and thus improved the enzymatic hydrolysis efficiency of polysaccharides substrate, and the increment was consistent with the amounts of hydroxyl and carboxyl groups in the pretreated lignin. MAMS pretreatment could effectively change the chemical structures of lignin and significantly reduce its negative effect on enzymatic hydrolysis of lignocellulosic biomass.

## Conclusions

For effective utilization of lignocellulosic biomass, pretreatment is a primary and essential procedure to destroy its recalcitrant structure and high crystallinity structure of cellulose. The combination of MA and metal salt (chlorides and nitrates of Al and Fe) significantly changed the recalcitrant structural characteristics of lignocellulosic matrix, and MA + AlCl_3_ pretreatment showed the best synergistic interaction effect. The inhibitory effect of lignin remarkably reduced after MAMS pretreatment ascribed to that the increased hydrophilicity weakened the binding force between enzymes and lignin and reduce the nonproductive adsorption of enzymes on lignin. MAMS pretreatment effectively overcame the biomass recalcitrance, including the complex and compact structure of lignocellulosic matrix, crystallinity of cellulose, and inhibition of lignin, thus enhanced the enzymatic conversion of lignocellulosic biomass without the removal of lignin.

## Methods

### Materials

SCB was obtained from Nanning Sugar Manufacturing Co., Ltd. (Guangxi, China). The sun-dried SCB was comminuted and screened through a standard sieve of 60 mesh size (250 μm). Sun-dried SCB (equilibrium moisture = 7%) contained 44.04% cellulose, 30.77% hemicellulose, 22.80% lignin, and 2.39% ash and others (dry basis), determined according to National Renewable Energy Laboratory (NREL) method. Commercial cellulase enzymes (65 filter paper units (FPU)/mL, containing β-glucosidase, exoglucanase, endoglucanase, and xylanase), glucose and xylose (chromatographically pure) were purchased from Aladdin Industrial Corporation (Shanghai, China). Metal salts and other chemical reagents were of analytical grade, used without further purification, and obtained from commercial sources. Deionized water was used throughout the work.

### Isolation of holocellulose from SCB

Holocellulose isolated from SCB was adapted from the reported procedure [53]: comminuted SCB (20 g) was added in a 1 L Erlenmeyer flask, and then 600 mL of water, 5 mL of acetic acid, and 6 g of NaClO_2_ were added and evenly mixed by stirring. The flask was covered and placed in a water bath at 75 °C. After 1 h, another 5 mL of acetic acid and 6 g of NaClO_2_ were added, and the mixture was evenly stirred, and then covered and placed in the water bath at 75 °C for 1 h. The same operation was carried out ten times, resulting in a total of ten additions (50 mL of acetic acid and 60 g of NaClO_2_). The flask was cooled in an ice bath. The mixture was filtered and washed with cool deionized water until the filtrate was neutral and then washed with absolute alcohol. After vacuum dried at 45 °C to constant weight, the resulting holocellulose was obtained. The lignin content in holocellulose was analyzed by klason analysis and was determined to be 1.6% [54].

### Isolation of lignin from SCB

Lignin was isolated from SCB according to the methods reported in the literatures [55–57] with some modifications, and the detailed procedures are as follows: comminuted SCB (10 g, equilibrium moisture = 7%) and 61.3 mL deionized water were added to a stainless steel reaction tube with 15% (w/w) solids loading, and SCB was pretreated at 190 °C for 20 min under pressure in order to keep the water in a liquid state. After liquid hot water (LHW) pretreatment, the material was washed with hot deionized water through a Buchner funnel, vacuum dried at 45 °C for 24 h, and stored in a sealed container until use. LHW-pretreated SCB was suspended in an sodium acetate buffer (pH = 4.8) in a rotary shaker at 150 rpm and 50 °C for 48 h with the enzymes loading of 30 FPU/g substrate and a solid loading of 5%. After enzymatic hydrolysis, the solid was separated by centrifugation and washed with buffer solution and deionized water. Then, the insoluble residue was extracted twice (24 h each time) with 80% aqueous dioxane (v/v) with a solid to liquid ratio of 1:20 (g/mL). All supernatants were collected and concentrated under reduced pressure, regenerated in acidic water (pH = 2). The regenerated solid was filtered and washed with deionized water until the filtrate was neutral. After vacuum dried at 45 °C to constant weight, the resulting lignin was obtained.

### MAMS and MA pretreatments

**Fig. 8:**
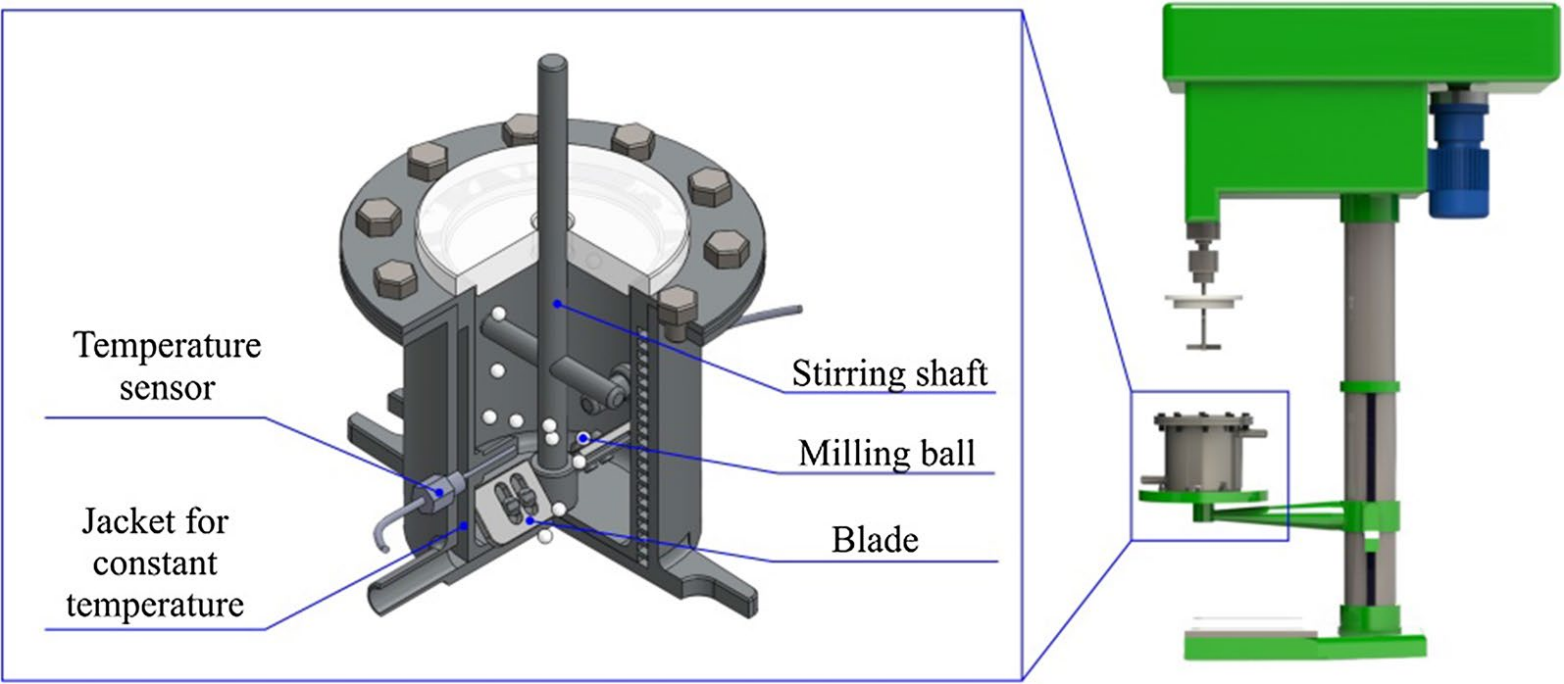
Schematic diagram of stirring ball mill

SCB, holocellulose, and lignin samples were subjected to MAMS and MA pretreatments, which were performed in a customized stirring ball mill (Fig. 8). For MAMS pretreatment, a fixed amount of milling balls (500 mL, 5 mm diameter) was first added into a jacketed stainless steel tank (1200 mL). Then, 20.0 g of the sample (dry basis) was mixed with a certain amount of solid-state metal salt (0.15 mmol/g dry sample). The mixture was put into the tank and subjected to dry milling at a speed of 500 rpm under a constant temperature of 50 °C by circulating the thermostatic water in the jacket of the tank. After milling for 60 min, the balls were removed from the milled sample by a sieve. The resulting sample without the removal of metal salt was vacuum dried at 45 °C for 24 h and then was stored in a sealed container for characterization and enzymatic hydrolysis.

MA pretreatment of the samples was operated by the same method as the MAMS pretreatment, without the addition of metal salts.

### Enzymatic hydrolysis

Enzymatic hydrolysis of SCB, holocellulose, and holocellulose + lignin (mass ratio of holocellulose to lignin was consistent with that in SCB) samples was conducted at 1.5% (w/v) in 10 mL of 0.05 M sodium acetate buffer (pH = 4.8) in a rotary shaker at 150 rpm and 50 °C with the enzymes loading of 12 FPU/g polysaccharides substrate (dry weight of cellulose and hemicellulose). After 48 h, 1 mL of hydrolysate was first filtered by a 0.22 µm microporous filter membrane and then was kept in boiling water for 1 min to inactivate the enzymes. Monosaccharides (mainly glucose and xylose) in the hydrolysate were quantified by high-performance liquid chromatography (LC-6AD, Shimadzu, Japan) using an Aminex HPX-87H column (Bio-Rad, Hercules, CA, USA) at 60 °C with 5 mM H_2_SO_4_ as the mobile phase and a refractive index detector (RID-10A, Shimadzu). Saccharification yields of the samples were measured by calculating the ratio of the determined amount of monosaccharides to the theoretically obtained monosaccharides.

### Characterization

XRD analysis of the SCB samples was performed by a D/MAX 2500 V diffractometer (Rigaku, Japan). XRD patterns were recorded from 5° to 40° with a speed of 0.02°. The determination was performed with Ni-filtered Cu K*α* radiation (*λ* = 0.154 nm) at 40 kV and 30 mA. Crystallinity index (CrI) was calculated by referring to diffraction intensities of crystalline area and amorphous region according to the following equation [58]:

2 $${\text{CrI}}(\% ) = \frac{{I_{\text{cryst}} - I_{\text{am}} }}{{I_{\text{cryst}} }} \times 100\%$$

where *I*_cryst_ is the diffraction intensity of the crystalline peak at about 2*θ *= 22°, and *I*_am_ is the diffraction intensity of the amorphous peak at about 2*θ *= 18°.

FTIR spectra of the samples were acquired on a Nicolet IS 10 Spectrometer (Thermo, USA) using the KBr disk technique. Dried samples (2 mg) were mixed and finely ground with KBr (200 mg). The spectra were obtained with a resolution of 4 cm^−1^ and a wavenumber range of 4000–400 cm^−1^.

XPS spectra of the samples were obtained by an X-ray photoelectron spectrometer (Thermo Fisher Scientific, USA) with a monochromatic Al K*α* (1486.6 eV) X-ray. The monochromatized Al K*α* X-ray source was operated in constant analyzer energy mode. A deconvolution curve fitting was performed for the C 1 s and the O 1 s peaks. The spectra were fitted using the Gaussian peak profiles and a linear background.

SEM analysis was performed by an S-3400 N scanning electron microscope (Hitachi, Japan). The samples were fixed on a sample bench using double-sided tape and then a thin layer of gold was coated on the samples prior to measurement to improve the conductivity. SEM micrographs were obtained to observe the surface morphologies of untreated and different pretreated SCB samples.

UV/vis spectroscopy analysis was conducted using a 2802 s UV–vis spectrometer (UNIC, USA) in the wavelength range 200–500 nm, at a slit width of 1 nm and a moderate scan velocity. About 0.015 g of the lignin sample was dissolved in 10 mL of water and a 1,4-dioxane mixture (volume ratio of water/1,4-dioxane = 1:9). Diluting 2.0 mL of the solution to 50.0 mL with a phosphate buffer (pH = 6) prepared the neutral solution, and diluting 2.0 mL of the solution to 50.0 mL with a sodium borate buffer (pH = 12) prepared the alkaline solution. The neutral spectra were determined by measuring the absorbance of the neutral solution. The reference was prepared according to the preparation of a neutral solution without the sample. The difference spectra were directly determined by measuring the absorbance of the alkaline solution relative to the neutral solution [49].

^1^H NMR spectra were accumulated on an AVANCE III HD 600 spectrometer (Bruker, Switzerland). 100 mg of lignin sample was dissolved in 1 mL of DMSO-d6 (99.9% deuterated, 0.05% tetramethylsilane) and left overnight in order to obtain complete dissolution. Trioxane was used as internal standard, and a certain amount of trioxane (mass ratio of trioxane:lignin = 1:10) was dissolved together with lignin for quantitative analysis. The solution was filtered with microporous filter membrane before measurement. ^1^H NMR spectra were accumulated at 600 MHz with a spectral width of 12,315.271 Hz with the cumulative scans of 1024 scans.

### Statistical analysis

All enzymatic hydrolysis experiments were carried out at least in triplicate, and the data were statistical average values. The significant differences among the panel data were analyzed by one-way ANOVA with Tukey’s test (*p* < 0.05) using SPSS software. The figures were plotted using Origin 8.0. Error bars were derived from the standard deviation of panel data.

## Additional file

**Additional file 1: Fig. S1.** XPS spectra of different SCB samples: (a) full-survey spectra, (b) peak fitting curves of C 1 s spectra, and (c) peak fitting curves of O 1 s spectra. **Fig. S2.** XPS spectra of different lignin samples: (a) full-survey spectra, (b) peak fitting curves of C 1 s spectra, and (c) peak fitting curves of O 1 s spectra. **Fig. S3.** UV/vis spectra of untreated and different pretreated lignin samples: (a) neutral spectra and (b) ionization difference spectra. **Fig. S4.** ^1^H NMR spectra of untreated and different pretreated lignin samples.

## Abbreviations

MA: mechanical activation
MAMS: mechanical activation + metal salt
SCB: sugarcane bagasse
DP: degree of polymerization
CrI: crystallinity index
XRD: X-ray diffraction
FTIR: Fourier transform infrared spectroscopy
XPS: X-ray photoelectron spectroscopy
SEM: scanning electron microscopy
UV/vis: ultraviolet/visible
FPU: filter paper unit
