# Supplementary material for: Overcoming biomass recalcitrance by synergistic pretreatment of mechanical activation and metal salt for enhancing enzymatic conversion of lignocellulose
Source: Biotechnol Biofuels. 2019 Jan 10;12:12. doi: 10.1186/s13068-019-1354-6 (PMC6327530; doi:10.1186/s13068-019-1354-6)
Supplement: Supplementary file 1 — Additional file 1: Fig. S1. XPS spectra of different SCB samples: (a) full-survey spectra, (b) peak fitting curves of C 1 s spectra, and (c) peak fitting curves of O 1 s spectra. Fig. S2. XPS spectra of different lignin samples: (a) full-survey spectra, (b) peak fitting curves of C 1 s spectra, and (c) peak fitting curves of O 1 s spectra. Fig. S3. UV/vis spectra of untreated and different pretreated lignin samples: (a) neutral spectra and (b) ionization difference spectra. Fig. S4. 1H NMR spectra of untreated and different pretreated lignin samples. [file 13068_2019_1354_MOESM1_ESM.docx]

**Additional file 1**

**Overcoming biomass recalcitrance by** **synergistic pretreatment of mechanical activation and metal salt for enhancing** **enzymatic conversion of lignocellulose**

Yanjuan Zhang^1,2^, Min Huang^1^, Jianmei Su^1^, Huayu Hu^1,2^, Mei Yang^1^, Zuqiang Huang^1,2*^, Dong Chen^2^, Juan Wu^1^ and Zhenfei Feng^1^

^1^ School of Chemistry and Chemical Engineering, Guangxi University, Nanning 530004, China

^2^ State Key Laboratory of Non-Food Biomass and Enzyme Technology, Guangxi Academy of Sciences, Nanning 530007, China

^*^ Correspondence: huangzq@gxu.edu.cn (Z. Huang)

**Fig. S1** XPS spectra of different SCB samples: (a) full-survey spectra, (b) peak fitting curves of C 1s spectra, and (c) peak fitting curves of O 1s spectra

**Fig. S2** XPS spectra of different lignin samples: (a) full-survey spectra, (b) peak fitting curves of C 1s spectra, and (c) peak fitting curves of O 1s spectra

**Fig. S3** UV/vis spectra of untreated and different pretreated lignin samples: (a) neutral spectra and (b) ionization difference spectra

**Fig. S4** ^1^H NMR spectra of untreated and different pretreated lignin samples
